# Presenting a case of a mucinous adenocarcinoma of an exstrophic bladder in an adult patient and a review of literature

**DOI:** 10.1186/1471-2482-13-S2-S36

**Published:** 2013-10-08

**Authors:** Giovanni Di Lauro, Fabrizio Iacono, Antonio Ruffo, Leo Romis, Salvatore Mordente, Umberto Pane, Ester Illiano, Giuseppe Romeo, Domenico Prezioso, Bruno Amato

**Affiliations:** 1 Hospital Santa Maria delle grazie', Via Domitiana località La Schiana - 80078 - Pozzuoli, Naples, Italy; 2 Department of Urology, University "Federico II" of Naples, Via Pansini, 5, 80131, Naples, Italy; 3 Department of Genaral, Geriatric, Oncologic Surgery and Advanced Technologies, University "Federico II" of Naples, Via Pansini, 5, 80131, Naples, Italy

## Abstract

**Background:**

Bladder exstrophy occurs in approximately 1 in 35,000 live births and is associated with an increased incidence of bladder cancer.

The primary mucinous adenocarcinoma of the bladder is an extremely rare urologic entity, which is found in less than 2% of all urinary bladder tumours and is often presented as metastatic. This is the first case in literature of a primary mucinous adenocarcinoma of an unreconstructed exstrophic bladder.

**Case presentation:**

A 55-year old male patient was diagnosed with a primary mucinous adenocarcinoma of an unreconstructed exstrophic bladder. Examination of the entire gastrointestinal tract shown there were not other primary cites. Immunohistochemistry confirmed the nature of the tumour. The patient underwent a radical cystoprostatectomy with en block bilateral pelvic lymphadenectomy, urinary diversion with a cutaneous ureterostomy and epidpadias repair.

**Conclusion:**

Adult bladder exstrophy and epispadia correction is a very rare practice in urology due the fact that this congenital disease is diagnosed and corrected in neonates. We advocate the radical surgical management, after exclusion of any primary malignant sites related to the gastrointestinal tract.

## Background

Bladder exstrophy is a complex congenital abnormality and its management is normally done in neonates.

Reports on exstrophy patients presenting in adulthood are very rare. Bladder exstrophy occurs in approximately 1 in 35,000 live births and is associated with an increased incidence of bladder cancer. Urinary bladder cancer is the second most frequent tumour of the genitourinary tract [1]. Adenocarcinomas of the bladder is an uncommon malignant neoplasm and account for less than 2% of all bladder cancers [2].

Few series have been reported in literature, but most contain small numbers of cases.

One of the most common forms of adenocarcinoma of the bladder is the metastatic adenocarcinoma. The primary sites for these tumours include the rectum, stomach, endometrium, breast, prostate, and ovaries.

The reported incidence among exstrophy cohorts varies from 3.3% to 7.5%, which is several times greater than in age-matched controls in the general population [3], [4].

In contrast, treatmentt of bladder exstrophy in patients presenting in adulthood is rarely reported in the literature [5], [6], [7], [8], [9]. Delayed presentation may be due to lack of awareness, ignorance, social embarrassment or even lack of appropriate facilities.

The etiology of this increased incidence remains unclear. The environmental stimuli and chronic infection may cause bladder cells to undergo glandular metaplasia [10]. After excluding patients who underwent reconstruction, only around 113 cases of cancer occurring in exstrophic bladders have been reported thus far. Approximately 90% were adenocarcinomas and 5% were squamous cell carcinomas[3], [4], [10], [11]. These cancers tend to be aggressive, and most patients undergo radical cystectomy as primary therapy[3], [4].

There are a variety of histological types among adenocarcinomas, including papillary, glandular, adenoid cystic, clear cell, mucinous and signet-ring cell carcinomas. The last of these types is characterized by the presence of intracellular vacuoles filled with mucin that displace the hyperchromatic nuclei [12]. This patient was diagnosed with the mucinous and signet-ring cell subtypes that is very rare and correspond to 20% of the bladder adenocarcinoma [12],[13].

## Methods

A 55-year-old man with classic bladder exstrophy who had never undergone a reconstruction earlier presented with a lesion of all his bladder [Figure 1]. The patient was otherwise healthy. His renal function tests were normal. The intravenous pyelogram showed bilateral normal excretory kidneys with no cystogram phase. The characteristic skeletal defects of exstrophy complex such as widening of the pubic symphysis caused by malrotation of the innominate bones in relation to the sagittal plane of the body along both sacroiliac joints were present. The metastatic work up was negative as seen on the CT scan. No preoperative bladder biopsy were performed.

We advocate the radical surgical management, after exclusion of any primary malignant sites related to the gastrointestinal tract.

The patient underwent radical cystectomy, closure of the infra-umbelical defect with Cardiff repair with On-lay mesh repair [14] and epispadias repair [Figure 3 - 4]

A wide local lymphadenectomy was performed [Figure 5].

Abdominal wall closure was done with a fasciocutaneous M-plasty [Figure 6 ].

Osteotomy was not done.

The orientation of the tumor on final clinical and pathologic examination in our patient showed that the bladder of 11x8 cm was completly occupied by a nodular, grey lesion of a maximum diameter of 10 cm producing mucin [Figure 2], thus strongly suggesting the bladder as the primary source.

## Results and discussion

Histopathological examination revealed a G3 poorly differentiated (High grade) mucin-producing adenocarcinoma, with intracellular and extracellular production of mucin. The lymph nodes examinated were negative showing a r*eactive lymphoid hyperplasia*. The skin margins were not involved. The patient was not given any adjuvant therapy and is doing well at six months of follow-up.

Other primary sites for the tumor had been excluded and, in the absence of digestive tract tumor and for confirmation that it was a primary bladder tumor, an immunohistochemistry study was performed.

Regarding immunohistochemistry, adenocarcinoma of the urinary bladder expresses CEA, CDX-2, MUC-1, MUC-2 and MUC-3, same as colonic adenocarcinoma. Cytokeratins 7 and 20 were positive, in contrast with colonic adenocarcinoma that expresses cytokeratin 20 but not cytokeratin 7 [15].

## Conclusions

Bladder exstrophy and epispadia is a very rare congenital disease in urology.

Because the vast majority of patients with bladder exstrophy now undergo reconstruction at a young age, unreconstructed bladder exstrophy in an adult patient is exceedingly rare.

We advocate the radical surgical management, after exclusion of any primary malignant sites related to the gastrointestinal tract.

With proper surgical management of late presenting bladder exstrophy patients, an acceptable functional and cosmetic outcome can be achieved.

## Competing interests

The authors declare that they have no competing interests.

## Authors' contributions

GdL: conception and design, interpetration of data, given final approval of the version to be published. AR: acquisition of data, drafting the manuscript, given final approval of the version to be published. LR: acquisition of data, drafting the manuscript, given final approval of the version to be published. SM: acquisition of data, drafting the manuscript, given final approval of the version to be published. UP: critical revision, interpretation of data, given final approval of the version to be published. FI: critical revision, interpretation of data, given final approval of the version to be published. EI: critical revision, interpretation of data, given final approval of the version to be published. DP: critical revision, interpretation of data, given final approval of the version to be published. GR: critical revision, interpretation of data, given final approval of the version to to published. BA :critical revision, interpretation of data, given final approval of the version to be published.

## Authors' information

GdL : Consultant in Urology at Hospital Santa Maria delle grazie, Pozzuoli, Naples, Italy. AR : Resident in Urology at Federico II University, Naples, Italy. LR : Resident in Urology at Hospital Santa Maria delle grazie, Pozzuoli, Naples, Italy. SM : Resident in Urology at Hospital Santa Maria delle grazie, Pozzuoli, Naples, Italy. UP : Resident at Hospital Santa Maria delle grazie, Pozzuoli, Naples, Italy. FI : Associate Professor of Urology at Federico II University, Naples, Italy. GR : Resident in Urology at Federico II University, Naples, Italy. EI : Resident in Urology at Federico II University, Naples, Italy. DP : Associate Professor of Urology at Federico II University, Naples, Italy. BA : Associate Professor of Surgery at Federico II University, Naples, Italy.

## Declarations

Funding for this article came from personal funds.

This article has been published as part of *BMC Surgery *Volume 13 Supplement 2, 2013: Proceedings from the 26th National Congress of the Italian Society of Geriatric Surgery. The full contents of the supplement are available online at http://www.biomedcentral.com/bmcsurg/supplements/13/S2

## References

[B1] Grossfeld GD Carroll PR Evaluation of asymptomatic microscopic hematuria Urol Clin North Am 1998 25 661 667 10026773 10.1016/s0094-0143(05)70055-0

[B2] Dahm P Gschwend JE Malignant non-urothelial neoplasms of the urinary bladder: a review Eur Urol 2003 44 672 681 14644119 10.1016/s0302-2838(03)00416-0

[B3] Smeulders N Woodhouse CR Neoplasia in adult exstrophy patients BJU Int 2001 87 7 623 8 11350401 10.1046/j.1464-410x.2001.02136.x

[B4] Paulhac P Maisonnette F Bourg S Dumas JP Colombeau P Adenocarcinomain the exstrophic bladder Urology 1999 54 4 744 10754144 10.1016/s0090-4295(99)00237-x

[B5] Matsuda T Okada Y Takeuchi H Adult female exstrophy of bladder treated with a Koch continent reservoir: report of a case Urol Int 1987 42 74 76 3590408 10.1159/000281857

[B6] Gulati P Yadav SP Sharma U Management of bladder exstrophy in adulthood: report of 2 cases J Urol 1997 157 947 949 9072609

[B7] Ozdiler E Sarica K Baltaci S A 49 year old woman with non complicated exstrophy of bladder Int Urol Nephrol 1996 28 747 750 9089040 10.1007/BF02550721

[B8] Quattara K Daffe S Timberly A Exstrophy of bladder in adults J Urol (Paris) 1992 98 64 65 1527404

[B9] Andankar Mukund G Pathak Hemant R Mahajan Ramesh Bladder preservation in adult classic exstrophy: early results of four patients Urology 2001 57 906 910 11337291 10.1016/s0090-4295(01)00959-1

[B10] McIntosh JF Worley G Adenocarcinoma arising in exstrophy of the bladder: Report of two cases and review of the literature J Urol 1955 73 5 820 9 14368728 10.1016/S0022-5347(17)67481-0

[B11] Bansal P Gupta A Mongha R Kundu AK Squamous cell carcinoma in exstrophic unreconstructed urinary bladder in an adult Saudi J Kidney Dis Transpl 2012 23 122 4 22237233

[B12] Fiter L Gimeno F Martin L Gómez Tejeda L Signet-ring cell adenocarcinoma of bladder Urology 1993 41 1 30 3 8380512 10.1016/0090-4295(93)90239-7

[B13] Zaghloul MS Nouh A Nazmy M Long-term results of primary adenocarcinoma of the urinary bladder: a report on 192 patients Urol Oncol 2006 24 1 13 20 16414487 10.1016/j.urolonc.2005.05.027

[B14] Shukla VK Mongha R Gupta N Chauhan VS Incisional hernia-comparison of mesh repair with Cardiff repair: An university hospital experience Hernia 2005 9 3 238 41 15902511 10.1007/s10029-005-0326-x

[B15] Bostwick DG Cheng L Urologic Surgical Pathology. Adenocarcinoma of the Urinary Bladder 2008 Elsevier, New York 300 302

